# The cardiovascular, metabolic, fetal and neonatal effects of CPAP use in pregnant women: a systematic review

**DOI:** 10.5935/1984-0063.20210024

**Published:** 2022

**Authors:** Debora Petrungaro Migueis, Arthur Urel, Camila Curado dos Santos, Andre Accetta, Marcelo Burla

**Affiliations:** 1 Federal Fluminense University, General and specialized surgery - Niterói - RJ -Brazil.; 2 Rio de Janeiro Federal University, Gynecology and obstetric - Rio de Janeiro - RJ - Brazil.

**Keywords:** Continuous Positive Airway Pressure, Obstructive Sleep Apnea, Gestational Diabetes. Preeclampsia, Pregnancy, Hypertension, Pregnancy-Induced

## Abstract

Continuous positive airway pressure (CPAP) is the standard treatment for obstructive sleep apnea (OSA), but its outcomes for the pregnant are still undefined. This study aims to review current CPAP intervention during pregnancy, discuss published trials, and propose relevant issues that have yet to be addressed satisfactorily about the cardiovascular, metabolic, fetal, and neonatal effects of CPAP treatment during gestation. Two authors independently conducted a systematic review until March 28th, 2021 on PubMed, BVS, and Cochrane Library, using PRISMA guidelines, and risk of bias. Discrepancies were reconciled by a third reviewer. Of 59 identified citations, eight original trials have submitted a total of 90 pregnant women to polysomnography and CPAP therapy. Four studies performed in samples with hypertension or preeclampsia presented blood pressure decrease or maintained the antihypertensive drug dose in the CPAP group. After CPAP utilization, one trial registered cardiac output and stroke volume increase with heart rate and peripheral vascular resistance decrease, which were correlated with birth weight increment. Others documented a higher Apgar in the CPAP group and more fetal movements during CPAP use. There was a reduction in serum uric acid and tumor necrosis factor-alpha in the CPAP groups whose blood pressure decreased. However, two weeks of CPAP use in women with gestational diabetes and OSA did not improve glucose levels but raised the insulin secretion in those adherents to CPAP. Despite these positive results without adverse effects, randomized controlled trials with standardized follow-up in larger populations are required to determine CPAP therapy recommendations in pregnancy.

## INTRODUCTION

Sleep-related breathing disorder (SBD) includes diverse pathologies: central sleep apnea syndrome, sleep-related hypoventilation disorders, sleep-related hypoxemia disorders, and obstructive sleep apnea (OSA), which is the most prevalent SBD^1^. OSA consists of partial or complete upper airway obstruction that leads to repetitive hypopnea or apnea, respectively^1^. Consequently, recurrent hypoxia and cortical arousals cause excessive diurnal sleepiness^2^ and quality of life impairment^3^. In individuals with OSA, the episodes of oxygen desaturation and arousals^1^ involve synergistic processes, including sympathetic activation, oxidative stress, and systemic infammation, with higher serum levels of catecholamines and proatherogenic mediators, such as interleukin (IL)-6, tumor necrosis factor-alpha (TNF-alpha), and C-reactive protein (CRP)^1,4,5,6^. The sympathetic nervous system activity and elevations in norepinephrine and epinephrine levels can raise blood pressure (BP) and heart rate, induce insulin resistance, inhibit pancreatic insulin secretion, and stimulate hepatic glucose release^4^. There is a significant association between SBD, decreased insulin sensitivity, and abnormal glucose mechanism independently of pregnancy^4,7,8^.

OSA pro-infammatory profile is associated with comorbidities, such as obesity, diabetes mellitus, hypertension, cardiac arrhythmia, stroke, coronary heart disease^9,10,11,12,13,14,15^, pulmonary edema, congestive heart failure^7^, and high mortality^14^. Previous studies assessed OSA intermittent hypoxia effects in the pregnant^7,16^. Chronic placental hypoxia was more common in OSA placenta than controls^16^. Placental tissue hypoxia may be correlated to the association between maternal OSA with growth restriction^7,16^ and low neonatal birth weight^8^. Also, OSA oxidative stress increases the risk of postoperative wound complications, hysterectomy, and intensive care unit admission^7^.

The treatment of choice for OSA is the continuous positive airway pressure (CPAP), which is associated with many improvements in adherent patients, such as alertness and BP control^14^. Recurrent hypoxia increases the degradation of adenosine triphosphatase into xanthine, which increases uric acid concentrations, whose level is associated with arterial hypertension^17,18^, endothelial dysfunction^19^, and a high risk of cardiovascular disease^20^. In support of this mechanism, the serum levels of pro-infammatory factors (TNF-alpha and uric acid) were significantly reduced in non-pregnant patients after 6 months of CPAP adherence for at least four hours per night, whereas they remained unchanged in those who exhibited poor compliance to CPAP therapy^21^. In non-pregnant samples with OSA, CPAP therapy improves sleepiness, quality of life, and also helps to lower BP in hypertensive patients with OSA^22^.

Although progesterone may be protective against OSA during pregnancy, other physiological changes contribute to the development or aggravation of this disorder, for instance, diaphragmatic elevation resulting in lower functional residual capacity, upper airway narrowing, edema^23^, gestational weight gain, enlargement of neck circumference^24^, fuid retention, and low sleep quality caused by increased urinary frequency and back pain^25^. OSA is a frequent disorder in pregnancy, with an estimated figure of 26.7% by the third trimester^24^, but often remains undiagnosed and untreated, leading to poor maternalfetal outcomes. Although CPAP is the standard treatment for OSA^14^, only a few trials have evaluated the outcomes of its use during pregnancy. The aim of this study is to review current CPAP use in pregnant women and to discuss published CPAP intervention related to gestation period, highlighting its outcomes for pregnancy, maternal comorbidities, fetal development, and neonatal analysis. Based on this review, we propose relevant issues that have yet to be clarified.

## MATERIAL AND METHODS

### Search strategy

A systematic review was carried out in the databases PubMed, Virtual Health Library (*BVS - Biblioteca Virtual de Saúde*), and Cochrane Library, according to the Preferred Reporting Items for Systematic Reviews and Metaanalysis (PRISMA) guidelines^26^. The MeSH terms included in the research were: [(“pregnancy” OR “pregnant women” OR “pregnant woman”) AND “obstructive sleep apnea” AND “continuous positive airway pressure”]; (“preeclampsia” AND “obstructive sleep apnea” AND “continuous positive airway pressure”); (“gestational hypertension” AND “obstructive sleep apnea” AND “continuous positive airway pressure”) as well as (“gestational diabetes” AND “obstructive sleep apnea” AND “continuous positive airway pressure”). The search terms also included a combination of the following keywords without MeSH terms: “gestational” AND “obstructive sleep apnea” AND “continuous positive airway pressure”. Each search was run separately and findings were merged. Only articles published in English until March 28^th^, 2021 (no lower date limit) were considered.

Initially, two reviewers (D.P.M. and A.U.) identified articles eligibility independently on March 28^th^, 2021 by the title and the abstract. Reference lists of original research and review articles were also examined to search relevant studies. The full text was retrieved if a decision could not be based on the abstract. Any disagreements were resolved through discussion between both, with adjudication by a third reviewer (C.C.S.) if it persisted. All possible effort was made to obtain data from authors, including contacting them by e-mail.

### Study eligibility criteria

The reviewers included original trials that have submitted pregnant women aged 18 years or older to a polysomnography and CPAP intervention. The study sample could be healthy, with OSA diagnosis or OSA risk factors (snoring, hypertension, preeclampsia, and gestational diabetes)^1^. The recommended sleep parameters according to the American Academy of Sleep Medicine (AASM) were required to detect the OSA severity, such as snoring, hypopnea, apnea, and the controversial respiratory effort related arousal (RERA)^1,27^. The apnea-hypopnea index (AHI) represents the number of apneas and hypopneas per hour of sleep. The respiratory disturbance index (RDI) consists of the number of apneas, hypopneas, and RERAs per hour of sleep^27^. The respiratory events index (REI) is calculated as the number of these events per hour of recording^27,28^. Mild OSA is diagnosed when RDI was 5 to 14.9, moderate OSA when RDI was 15 to 30, and severe OSA for RDI greater than 30^22^. Maternal comorbidities and the number of respiratory events measurement in polysomnography were used as comparative parameters between studies.

The following PICO questions (the acronym standing for patient, population or problem, submitted to a specific intervention or exposure, after which a defined comparison is performed on specified outcomes) were decided before the review process. The included articles had to address at least one of the following questions:

Does CPAP utilization during pregnancy improve somnolence and sleep quality questionnaires, maternal comorbidities, metabolic profile, fetus movements, and heart rate?

What are the effects of CPAP use by pregnant women on neonatal outcomes (Apgar score, birth weight, preterm delivery, unplanned cesarean, and intensive care admission)?

### Exclusion criteria

Studies in animals, men, or non-pregnant populations, case reports or case series with less than 5 subjects, screening tests accuracy, systematic reviews, letters, editorials, genetic aspects or basic/experimental research, trials in recruitment or analysis phases without published outcomes, and duplicated studies were excluded.

### Quality assessment

Two independent reviewers assessed the risk of bias and precision for each selected study using NIH guidance, a validated tool with specific instructions for assessing the internal validity of intervention studies with and without a control group, which consist of a 14-item for randomized clinical trial and a 12-item for pre-post intervention trial respectively^29^. Possible responses to each item were: “yes” meaning “low risk” of bias, “no” meaning “high risk”, and “cannot determine”, “not applicable”, or “not reported” grouped into “unclear risk” of bias. After the agreement of 2 authors (A.U. and D.P.M.) about each NIH item, this tool provided the risk of bias outcomes. Discrepancies were resolved by consensus in discussion with a third reviewer (C.C.S.).

### Extraction and synthesis of data

Afterwards, the available data were extracted from the included studies according to the subsequent topics.

#### Methodological issues

The following data were extracted from all the selected studies: main author; year of publication; the country where the study was performed; study design; the total number of patients included; mean age; maternal mean BMI; patients’ comorbidities; polysomnography type; and the criteria used to determine its parameters in all publications.

#### Objective and subjective sleep analysis

Polysomnography is regarded as the recommended standard for SDB diagnosis^1^. In the selected studies the following methodological questions were considered to ensure the comparability of obstructive respiratory events (apnea, hypopnea, and RERA)^1,28^ across the studies: 1) polysomnography types; 2) criteria used to score the sleep.

Moreover, subjective sleep quality analyses were accessed if available, using the following validated questionnaires during prenatal care: Epworth somnolence scale (ESS) and Pittsburgh sleep quality index (PSQI). These parameters were compared between control and CPAP groups as well as non-controlled pre-post CPAP studies.

The ESS is an eight-item questionnaire with situations to be classified by the patient on a scale from zero (no chance of falling asleep) to three (high chance of dozing off), intended to quantify daytime sleepiness^30^. A score higher than nine defines excessive diurnal somnolence^31^. Its use is recommended for OSA evaluation and follow-up^22^.

The PSQI evaluates sleep quality over a one-month period, comprising 19 items that quantify subjective sleep quality, sleep latency, sleep duration, habitual sleep efficiency, sleep disturbances, use of medication, and daytime dysfunction. Each item is represented on a 0-3 scale. Its final global score ranges from 0 to 21, and lower scores denote a healthier sleep quality^32^. This instrument has already been used by many researchers in pregnant samples^33^.

#### Analyzed variables of CPAP outcomes in the pregnant patients and their fetus

The analysis included polysomnography parameters, days of CPAP use, and the following p-values for comparison of control group or pre-post CPAP treatment, when available: non-invasive BP measured in rest, cardiovascular effects (cardiac output, heart rate, stroke volume, ejection time, peripheral vascular resistance), metabolic effects (serum levels of glucose, insulin, uric acid, TNF-alpha, CRP, and IL-6), fetal movements, neonatal outcomes (Apgar score, birth weight, preterm delivery, unplanned cesarean, and intensive care assistance).

#### Blood pressure and cardiovascular analysis

BP was measured once in rest at each visit, or the 24-h BP recording obtained every 30 minutes. CPAP therapy can improve the nocturnal BP levels or restore the physiologic nocturnal BP dip^34^ in OSA patients^14^.

Ejection time (ET), heart rate (HR), stroke volume (SV), cardiac output (CO), and total peripheral resistance (TPR) were registered with a non-invasive finger arterial photoplethysmography, using the Beatscope software and Modelflow method, that recorded continuously for the whole study, placed on the third and fourth finger on the left hand and alternated every 30 minutes^35^. Photoplethysmography can measure a range of features of the pulse, including the pulse transit time (PTT) of the arterial pulse wave, which can estimate arterial BP^36^. This is achieved by detecting the pulse wave at one body site location and measuring the time takes for it to reach a second distal location^36^. Heart rate variability analysis may indicate a predominant involvement of the sympathetic or parasympathetic tone in SBD, but its parameters (high and low frequency band) should be calculated by integrating the power spectral density in the respective frequency bands^37,38^ to be a reliable data.

#### Metabolic profile analysis

Blood collected from the participants was tested for uric acid^15,39^, CRP, TNF-alpha, and IL-6^15^. These pro-infammatory markers reflect oxidative stress in OSA patients during pregnancy or not^6,40,41^ and their levels decrease significantly after CPAP therapy^5,21^. Serum uric acid is a controversial predictor of maternal and fetal complications in women with pre-eclampsia^17,41^ and is not an OSA biomarker alone^18^. Nonetheless, it was correlated with BP, AHI, desaturation time, and index in a population-based survey^18^. Peripheral blood was collected and tested the same day for uric acid and CRP in mg/dL^15^. A tube of blood was prepared and stored at -80° centigrade until study completion. Serum levels of TNF-alpha and IL-6 were determined in pg/mL, using standard capture ELISA assays, with matched capture and specific detection antibodies and recombinant protein standards^15^.

Glucose tolerance was measured by fasting plasma glucose (FPG) and area under the curve (AUC) of glucose response (calculated using the trapezoidal rule) to meal tolerance test (MTT) before and after the 2 weeks randomization period. Homeostatic model assessment of insulin resistance (an index of fasting insulin resistance), the insulinogenic index (an estimate of early insulin secretion), and the Matsuda index (an index of whole-body insulin sensitivity) were calculated. The disposition index, an indicator of beta-cell function adjusted for insulin sensitivity, was calculated as a product of the insulinogenic index and the Matsuda index^10^.

### Fetal or neonatal analysis

The fetal activity sensors consisted of shallow aluminum cylinders, with a film of piezoelectric bi-layer plastic, stretched to form the equivalent of a stethoscope diaphragm placed on all quadrants of the maternal abdomen. Its signal was amplified by an AMLAB instrumentation computer. Profusion PSG software calculated the total number of fetal movements registered by sensors during the study night and fetal movements were not scored if they occurred in association with maternal movements^42^. Fetal heart rate is a marker of fetal well-being^42^ that can be captured by ultrasound signal from abdominal bands^43^ or cardiotocographic monitoring^44^.

Preterm delivery, birth weight, Apgar score, unplanned cesarean section, and neonatal intensive care admissions were also documented. The Apgar score was performed on newborns at one and five minutes after birth evaluating five criteria (skin color, heart rate, muscle tone, breathing effort, and irritability reflex) on a scale from zero to two. Scores ranging from 7 to 10 are associated with better newborn health conditions^45^.

## RESULTS

### Design and general characteristics of the included studies

**Figure 1.**
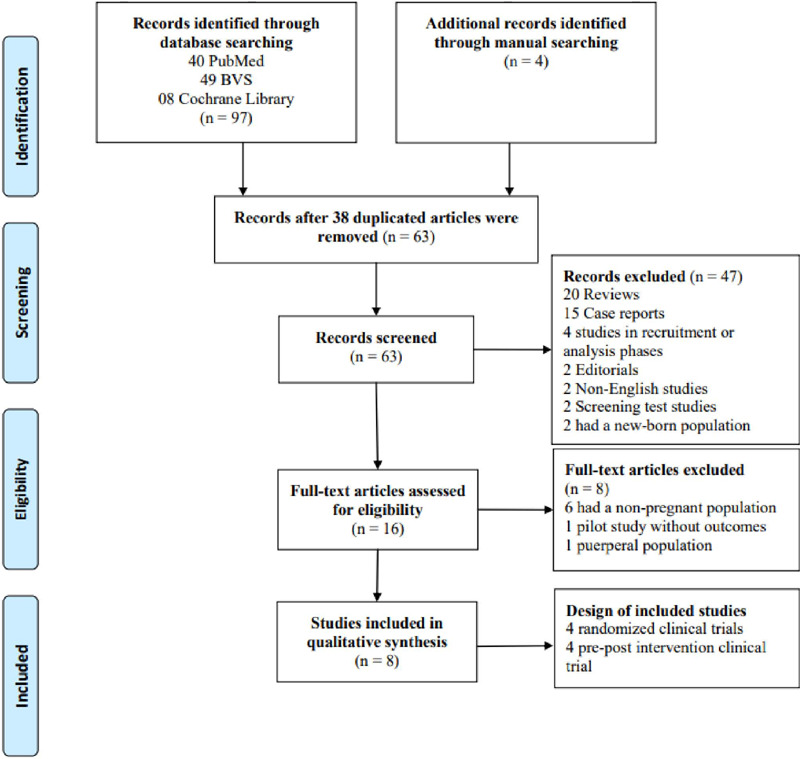
Flowchart for the included articles.

In March 2021, we identified 59 studies through database searching (40 PubMed, 49 BVS, and 8 Cochrane Library) using the keywords according to PRISMA statement and 4 studies by manual search. After duplicate removal, 63 articles were screened, whereas 47 were excluded: 20 reviews, 15 case reports, 2 non-English publications, 2 new-born populations, 4 studies in recruitment or analysis phases, 2 screening test analyses, and 2 editorials. Then, 16 studies were selected for a detailed full reading evaluation, but 8 did not fill the including criteria: 6 had a non-pregnant population 1 postpartum article, and 1 pilot study without published outcomes. Hence, the final systematic review included eight articles (Figure 1).

**Figure 2.**
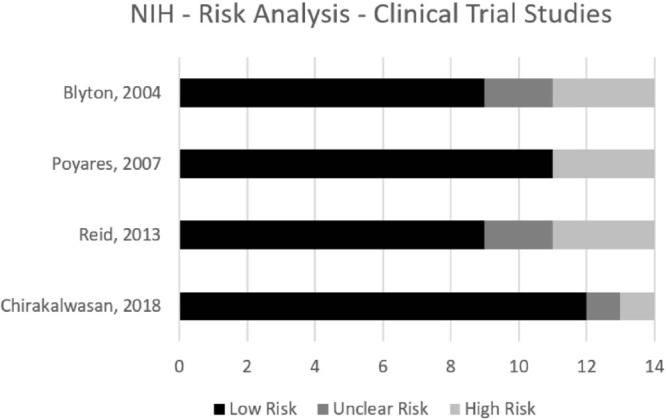
NIH - Randomized Clinical Trial; Risk of bias summarized for all the included studies. Results in the graph show the level of risk of bias (%) as high, unclear, or low risk.

**Figure 3.**
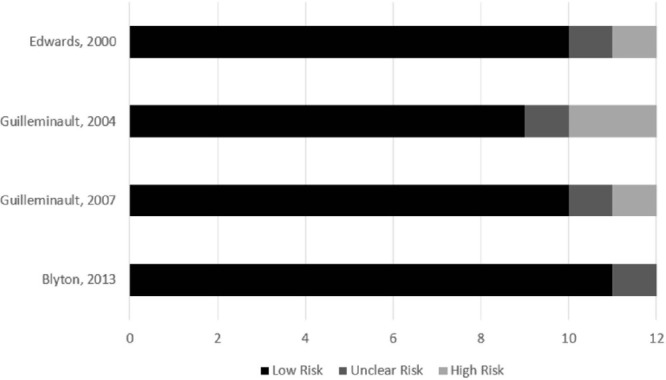
NIH - Pre and Post Intervention Clinical Trial; Risk of bias summarized for all the included studies. Results in the graph show the level of risk of bias (%) as high, unclear, or low risk.

The eight remaining studies were read by two independent reviewers who assessed the risk of bias using NIH guidance. The customization of the NIH quality assessment is presented as percentage in Figures 2 and 3. Despite all studies being clinical trials, only four of them (50.0%) are randomized controlled trials^10,12,15,35^, while four studies (50.0%) compared the same group pre-post CPAP therapy^34,39,42,46^. All studies used polysomnography to determine SDB and reached a low-risk score of 50% or higher, according to NIH. The majority of the studies described the populations in detail, but none of the trials blinded the participants with sham CPAP, whose airway pressure does not treat apnea or hypopnea, but may interfere with sleep quality and duration^47^.

The study design, population characteristics, and sleep study of the selected trials for this review are shown in Table 1. Although each study had small-sized samples, this systematic review combined for the frst time a total of 90 pregnant women who underwent CPAP therapy. None of them were multicentric, all participants were handpicked from a prenatal care service, or were already in treatment with the sleep medicine department of the same hospital or clinic.

**Table 1.** Selected studies.

Author, [reference], publication year	Country	Clinical trial	CPAP group (N)	Control Group (N)	Gestational age during CPAP use (weeks)	Mean age (years old) (range)	Mean BMI (kg/m^2^) (range)	Maternal comorbidities	Polysomnography type; the criteria used for score
Edwards et al. (2000)^39^	Australia	non-RCT	11	--	35±1	34±2	27±1 (24-30)	Preeclampsia	Type 1; Rechtschaffen and Kales (1968).
Blyton et al. (2004)^35^	Australia	RCT	12	15 without preeclampsia 12 with preeclampsia	24-38	33±6 (24-39)	30.5±4.7 (23-40)	Preeclampsia	Type 2; Rechtschaffen and Kales (1968).
Guilleminault et al. (2004)^46^	USA	non-RCT	12	--	4-32	28.4 (24-33)	24.03 (22.4–26.2)	OSA diagnosis	Type 1; AASM 1999.
Guilleminault et al. (2007)^34^	USA	non-RCT	12	--	5-36	29±3	3 Obese: 32.1±1; 7 Hypertension: 24.8±1.8; 2 Prior preeclampsia: 24.2±0.4	Pregnant women with preeclampsia risk factors	Type 1; AASM 1999.
Poyares et al. (2007)^12^	Brazil	RCT	7	9	17-35	32.8±7.0	24.3±1.7	Hypertension and chronic snoring	Type 1; AASM 1999.
Blyton et al. (2013)^42^	Australia	non-RCT	10	--	27-37	30.1 (20-35)	N.A.	Preeclampsia	Type 2; Rechtschaffen and Kales (1968).
Reid et al. (2013)^15^	Canada	RCT	11	13 MAD/Nasal Strip	34±3	30.27±5.53	32.97±7.36	Gestational hypertension	Type 2; AASM 2007.
Chirakalwasan et al. (2018)^10^	Thailand	RCT	15	17	24-34	31.6±6.0	30.8±3.6	Gestational diabetes	Type 4; Watch-PAT 200.

Notes: Randomized controlled trial (RCT); Non-randomized controlled trial (non-RCT); Obstructive sleep apnea (OSA); Mandibular advancement device (MAD); Continuous positive airway pressure (CPAP); American Academy of Sleep Medicine (AASM).

Three of the eight selected studies (37.5%) belongs to the same research group in Australia, led by Blyton and Edward^34,39,42^, two of Guilleminault’s publications (25%) were from the United States of America^34,46^, one trial was from Brazil^12^, one was conducted in Thailand^10^, and another in Canada^15^. The publication year ranged between 2000^39^ and 2018^10^ (Table 1). The patients’ mean age ranged between 29±3^46^ and 34±2 years-old^39^. Maternal mean BMI varied between studies, three studies (37.5%) had mean BMI in eutrophic range^12,34,46^, one study (12.5%) in overweight range^39^, and three studies (37.5%) in class 1 obesity range^10,15,35^ (Table 1). Comorbidities or risk of hypertension and preeclampsia were analyzed in six of the selected studies (75.0%)^12,15,34,35,39,42^, and gestational diabetes in one of them (12.5%)^10^. The remaining study (12.5%) was composed of healthy pregnant women with snoring, suspected OSA, or confrmed diagnosis of OSA^46^ (Table 1).

### Analyzed variables in each study

Two tables summarized the extracted data according to women’s comorbidities: Table 2 describes participants with or at risk for preeclampsia, while Table 3 includes pregnant women with the following characteristics: gestational diabetes or OSA diagnosis.

**Table 2.** Trials in pregnant with hypertension, with or at risk for preeclampsia.

Author, [reference], publication year	Mean AHI/RDI pre-CPAP (/hour)	CPAP use (days)	Mean blood pressurepre, post-CPAP (*p*-value)	Cardiovascular effectspre, post-CPAP (*p*-value)	Metabolic effectspre, post-CPAP (*p*-value)	Fetal or neonatal analysis	Subjective analysis with ESS, PSQI (*p*-value)
Edwards (2000)^39^	RDI 5±1	One night	Reduced during the night with CPAP treatment. SBP (*p* =.012) DBP (*p*=.007)	Heart rate did not change	Serum uric acid was reduced in CPAP use (*p*=0.006)	N.A.	N.A.
Blyton et al. (2004)^35^	RDI 22±23	One night	Decreased by 3±3 from wakefulness to sleep (*p*=0.005)	During sleep in CPAP subjects: The decrement in cardiac output was reversed (*p*=0.03) The increased hearth rate was reversed (*p*=0.01) Stroke volume increased during sleep (*p*=0.004) Ejection time increased and normalized (p=0.04) The increase in total peripheral resistance was attenuated (*p*=0.002)	N.A.	There was a significant correlation between birth weight and cardiac output (*p*<0.001).	N.A.
Guilleminault et al. (2007)^34^	RDI 8.5±2.6	All the pregnancy	All seven women with chronic hypertension had no significant BP increase with CPAP and anti-hypertensive adjustment was not necessary.	The 24-h BP recording between 34 and 36 weeks of gestation kept the nocturnal BP dip.	N.A.	2 women with obesity and 1 with preeclampsia delivered prematurely and their infants required hospitalization.	N.A.
Poyares et al. (2007)^12^	AHI 3.1±1	Every day for at least one month	Control group needed an increased dose of antihypertensive medication. BP was significantly higher at control group at 35 weeks of gestation DBP (*p*=0.0003); SBP (*p*=0.001).	N.A.	N.A.	Apgar higher in the CPAP group (*p*=0.04). There was no significant difference in the birth weights.	N.A.
Blyton et al. (2013)^42^	AHI 7.0±1.8	One Night	N.A.	N.A.	N.A.	The number of fetal movements increased with CPAP use (*p*<0.0001).	N.A.
Reid et al. (2013)^15^	RDI 10.73±17.07	One Night	No difference in blood pressure between the CPAP and MAD groups (*p*=0.371)	N.A.	TNF-alpha decrement in the 9 women with blood pressure improvement (*p*=0.024). Other pro-inflammatory markers showed no difference.	N.A.	There was no significant difference between other baseline subjective analyses (ESS, *p*=0.946), (PSQI, *p*=0.362).

Notes: Apnea hypopnea index (AHI); Respiratory disturbance index (RDI); Blood pressure (BP); Systolic blood pressure (SBP); Diastolic blood pressure (DBP); Mandibular advancement device (MAD); Visual analogue scale (VAS); Pittsburgh sleep quality index (PSQI); Epworth sleepiness scale (EES); Continuous positive airway pressure (CPAP); Tumour necrosis factor alpha (TNF-alpha); N.A.: Not available data.

**Table 3.** Randomized controlled trial in pregnant without hypertension or preeclampsia.

Author [reference], publication year	Comorbidities	Mean AHI /REI pre-CPAP	CPAP use	Blood pressure or cardiovascular effects	Metabolic effects	Fetal or neonatal analysis	Subjective analysis with ESS, PSQI
		(range) (/hour)	(days)	(*p*-value)	(*p*-value)		(*p*-value)
Guilleminault et al. (2004)^46^	OSA diagnosis	AHI 21 (9-31)	All the pregnancy	N.A.	N.A.	There was no preterm delivery. All infants were healthy. Apgar scores were all above 8.	ESS decreased (*p*=0.0001)
Chirakalwasan (2018)^10^	Gestational diabetes	9.4 (interquartile range 6.4-12.4)	≥14 days	N.A.	Disposition index (pancreatic function) CPAP group 3.4±0.9; Control group -0.4±0.4; (*p* =0.002) Serum glucose (AUC) CPAP group -72.0±74.0; Control group 16.6±33.3; (*p*=0.344).	Those using CPAP longer than 2 weeks were less likely to have preterm delivery (p=0.002), neonatal intensive care unit/special care nursery admissions (*p* <0.001). No difference in birth weight and Apgar score.	N.A.

Notes: Obstructive sleep apnea (OSA); Apnea hypopnea index (AHI); Respiratory event index (REI); Area under the curve (AUC); Systolic blood pressure (SBP); Diastolic blood pressure (DBP); Pittsburgh sleep quality index (PSQI); Epworth sleepiness scale (ESS); Continuous positive airway pressure (CPAP); N.A.: Not available data.

### Subjective and objective sleep outcomes

Although all studies performed sleep studies once, there were different types of polysomnography and scoring criteria. Four selected studies (62.5%) performed type 1 polysomnography^12,34,39,46^, three trials (25%) used type 2^15,35,42^, and only one (12.5%) conducted type 4 polysomnography^10^. Regarding the scoring criteria, three selected studies (37.5%) used Rechtschaffen and Kales (1968)^35,42,39^, three (37.5%) used the AASM (1999) criteria^12,34,46^, one used the AASM (2007) criteria^15^, and the trial that used the validated type 4 polysomnography^48^ defined the respiratory event as a drop of the PAT signal amplitude with =3% oxyhemoglobin desaturation or an arousal (acceleration in the pulse rate or increase in wrist activity)^10,48^. Five selected studies (62.5%) measured OSA gravity using the respiratory disturbance index (RDI)^10,15,34,35,39^ as a parameter for respiratory events during sleep, in contrast, three other selected studies (37.5%) reported the apnea-hypopnea index (AHI)^12,42,46^. Nevertheless, regarding the results of sleep analysis pre CPAP usage, only one selected study (12.5%) had mean AHI<5^12^, fve of them (62.5%) presented with mean AHI/ RDI between fve and 14.9^10,15,34,39,42^, and two (25%) registered the mean AHI/RDI equal or higher than fifteen^35,46^.

From the eight selected studies for this review, only two (25%) analyzed the subjective sleep quality with the Epworth sleepiness scale (ESS) or the Pittsburgh sleep quality index (PSQI) during prenatal care in each physician appointment^15,46^. One RCT (12.5%) described ESS decrease in CPAP treatment compared with the same group before CPAP use^46^ and another trial (12.5%) reported no baseline difference in ESS and PSQI between CPAP therapy group compared with mandibular advancement device (MAD) + nasal strip treatment group^15^.

### CPAP usage time

Concerning CPAP usage time, none of the selected studies specified mean time use per night. Four of them (50%) registered the outcomes after CPAP therapy for one night^15,35,39,42^, while three studies (37.5%) underwent CPAP treatment for at least one month^12,34,46^, and another trial (12.5%) for at least two weeks^10^.

### Blood pressure and cardiovascular outcomes

Non-invasive BP was measured during different gestational ages (Table 1). For instance, Edwards et al. (2000)^39^ and Blyton et al. (2004)^34^ verified it during the polysomnography nights performed after 20 weeks of gestational age^35,39^, while Guilleminault et al. (2004)^46^, Guilleminault et al. (2007)^34^, and Poyares et al. (2007)^12^ did it during all prenatal care.

Of the eight selected studies for this review, five of them analyzed differences in BP between the CPAP and the control groups or before and after CPAP use^12,15,34,35,39^. There was no difference in BP levels between the CPAP treatment group and the MAD + nasal strip group^15^. This was the only trial performed in healthy pregnant women.

Five selected studies (62.5%) analyzed the outcomes of CPAP therapy in samples with or at risk for preeclampsia^12,34,35,42,39^ and four of them investigated the CPAP effects on BP^12,34,35,39^. In two studies there was a significant BP decrease after CPAP use compared with the control group or before CPAP treatment in the same group^35,39^. One reported the increase of BP medication in the control group, while the CPAP group maintained the same anti-hypertensive dose throughout all pregnancy^12^. Guilleminault et al. (2007)^34^ showed no significant BP increase with a combination of antihypertensive medication and CPAP during pregnancy in those with a history of chronic hypertension. Besides, the 24-h BP recording between 34 and 36 weeks of gestation kept the physiologic nocturnal BP dip in the entire population with risk factors for preeclampsia^34^.

Moreover, one of the eight selected studies (12.5%) analyzed the cardiovascular outcomes in 15 nulliparous controls and twenty-four women with severe preeclampsia randomized into two groups: with and without CPAP therapy^35^. This RCT showed an increased cardiac output, ejection time, and stroke volume, while heart rate and total peripheral vascular resistance decreased after CPAP use compared with the control group or before CPAP therapy^35^. However, Edwards et al. (2000)^39^ measured no significant change in maternal heart rate. None of the included articles analyzed maternal heart rate variability.

### Metabolic outcomes

Three trials (37.5%) from the eight selected studies monitored metabolic outcomes^10,15,39^. Chirakalwasan et al. (2018)^10^ described an improvement in the insulin secretion and pancreatic function after two weeks of CPAP use compared with the control group, although the mean changes of AUC, FPG, and serum glucose response to MTT showed no significant difference between both groups. Another trial registered a lower serum tumor necrosis factor alpha (TNF alpha) in the pregnant with BP improvement during CPAP treatment, yet other pro-infammatory markers (uric acid, CRP, and IL-6) showed no difference after CPAP therapy^15^. In contrast, Edwards et al. (2000)^39^ reported a significant decrease in serum uric acid in the women with preeclampsia whose BP reduced during CPAP utilization.

### Fetal or neonatal outcomes

Of the eight selected studies for this review, four of them (50%) analyzed fetal or neonatal outcomes^10,12,35,42^.

In participants with gestational diabetes, the CPAP treatment group was less likely to have a preterm delivery, unplanned cesarean section and neonatal intensive care unit/special care nursery compared with the group without CPAP use, but there was no difference in birth weight or Apgar score between both groups^10^.

In samples with preeclampsia^12,35,42^, one study reported higher Apgar scores for newborns of those who used CPAP compared with no CPAP treatment, despite no difference in birth weight^12^. However, another trial associated the maternal cardiovascular outcome improvement with birth weight in the CPAP group fetus^35^.

Finally, the last one reported more fetal movements in the same group of pregnant women during sleep with CPAP than without it^42^. None of the included studies analyzed fetal heart rate during CPAP monitoring.

## DISCUSSION

CPAP is the gold standard treatment for OSA and other SBD^14,22,49^. None of the studies reported CPAP adverse effects in pregnant women or neonatal participants. Besides, it is a safe treatment with long term benefts for OSA patients^50,51^, showing a strong correlation with improvement in cardiovascular functions^52^, BP^53,54,55^, diabetes^56,57,58^, infammatory markers^53,59,60^, subjective sleep quality, quality of life^60,61,62^, and mortality^63,64^. Although physiological changes in pregnancy contribute as risk factors for OSA’s development due to lower respiratory function, upper airway edema, and elevated estrogen^24,25^, the prevalence and comorbidities related to OSA in the pregnant are still a growing area of study. Many studies demonstrated the increasing prevalence of OSA during pregnancy, especially in the third trimester^24,65^. Despite its undeniable relevance, there were only eight clinical trials about CPAP treatment in pregnant population and its effects on maternal comorbidities (hypertension, preeclampsia, gestational diabetes, and OSA)^10,12,15,34,35,39,42,46^, subjective aspects (sleep quality, daytime sleepiness)^15,46^, fetal, and neonatal outcomes^10,12,34, 35, 42,46^.

Observational studies have linked hypertensive disorders of pregnancy and SDB^25^. A significant BP decrease was reported by the majority of the selected studies that analyzed BP in the CPAP treatment group^12,35,39^. CPAP may reduce intermittent hypoxia, oxidative stress, and endothelial dysfunction^23,25^. Alternatively, CPAP may decrease edema in the upper airway associated with preeclampsia^23,25^. Only the study whose control participants utilized MAD instead of no interventions reported no statistical difference in BP between the CPAP therapy group and the control group^15^. Possibly the use of these devices for a single night failed to demonstrate a significant BP decrease in women with gestational hypertension, and further RCTs with larger samples in similar gestational ages and longer follow-ups are necessary.

After CPAP treatment, the studies in the preeclampsia population registered an improvement of the BP^12,35,39^, cardiovascular outcomes^35^, serum uric acid levels^39^, the number of fetal movements^42^, and neonatal birth Apgar score^12^. Its pathogenic mechanisms share similarities with those underlying cardiovascular consequences of OSA, including ischemia-reperfusion injury, oxidative stress, and endothelial dysfunction^66,67^. This dangerous condition is associated with maternal complications, such as hypertension, proteinuria, edema, placental hypoperfusion, fetal complications (poor growth, prematurity), and maternal death^66^. Thereby, preventing preeclampsia, early diagnosis, and strict BP control are vital to reduce morbimortality. This systematic review supports the great benefits of CPAP use, with significant improvement BP in women with preeclampsia, but definitive conclusions about cardiovascular effects of CPAP therapy during pregnancy still require clinical trials in larger populations.

Two studies addressed maternal heart rate without heart rate variability parameters^35,39^, which impair its accuracy to evaluate autonomic system activation during pregnancy. In contrast, although fetal heart rate monitoring has been used during maternal sleep by previous investigators^43,68,69^, none of the selected studies analyzed it during CPAP monitoring. Therefore, further researches about maternal hypoxic conditions may use this feasible tool.

Four studies presented conflicting CPAP effects on fetal and neonatal outcomes^10,12,35,42^. Firstly, Blyton et al. (2013)^42^ correlated a higher number of fetal movements with the oxygen desaturation reduction during CPAP use than without it. Fetal movements are a measure of fetal well-being, which may be impaired by inspiratory airflow limitation during sleep in preeclampsia and reversed by CPAP intervention^42^. Regarding the Apgar score, one study reported^12^ significantly higher scores in the CPAP treatment group, while the other registered no statistical difference between both groups^10^, possibly because it is affected by many maternal-fetal factors, including preeclampsia, gestational age, and congenital malformations^45^. The Apgar score alone does not predict neonatal mortality, but it quantifies neonatal depression and provides the newborn status immediately after birth and the response to resuscitation if needed^45^. Only a RCT in gestational diabetes analyzed pre-term delivery and neonatal intensive care demand, detecting its improvement in the CPAP treatment group, despite no differences in birth weights between both groups^10^. There was another inconsistency regarding the birth weight. A study that conducted CPAP treatment in a population with hypertension and snoring during the whole pregnancy showed no difference between the CPAP and control groups^12^, while Blyton and et al. (2004)^35^ correlated CPAP treatment during only one night in a preeclampsia population without snoring with the improvement of maternal cardiac output during sleep and higher birth weight. Although preeclampsia is a risk factor for fetal growth restriction, birth weight is a multifactorial condition and neonatal outcome needs further research. The difference of protocols and follow up impair the comparative analysis, using unspecific parameters, such as birth weight and Apgar score.

Three selected studies^10,15,39^ that measured metabolic outcomes presented a decrease in pro-infammatory profile, despite different parameters analysis. In a sample without obesity, Edwards et al. (2000)^39^ associated the CPAP use with the decrease in serum uric acid, a prognostic marker for preeclampsia. However, the literature is unclear about the role of uric acid as a predictor of maternal and fetal complications, because it can be related to endothelial dysfunction, pro-infammatory effects, higher B P, and renal lesions in women with preeclampsia^17,70^, but this trial did not provide birth data to further analysis. Reid et al. (2013)^15^ registered a decrement in TNF-alpha after CPAP treatment in those whose BP decreased. Nevertheless, there was no significant difference in serum levels of CRP, uric acid, and IL-6 between CPAP and control group, possibly because their mean BMI was higher than 30kg/m^2^. IL-6 influences CRP production by the liver and is synthesized by adipose tissue in individuals with obesity^6^. These pro-infammatory markers present high levels in OSA patients^5,6,40^ and CRP had already been associated with obesity in subjects with OSA^71^. Oxidative stress raises their levels, which have been linked with hypertension and atherosclerosis^5,6,18,72^. Thus, there is a need for investigations to clarify the role of BP and obesity in these biomarkers. Also, intermittent hypoxia is associated with selective activation of infammatory pathways, and literature provides evidence of a correlation between these inflammatory markers and pathophysiology of cardiovascular complications in OSA patients, including coronary artery disease, congestive cardiac failure, and stroke^73,74,75^.

Chirakalwasan et al. (2018)^10^ determined an improvement in disposition index, suggesting an increase in the insulin sensitivity and pancreatic endocrine cells function, but AUC of serum glucose response did not exhibit a difference between CPAP and control groups. Repetitive cycles of hypoxemia with reoxygenation in SBD may initiate a cascade of biochemical reactions that increase oxidative stress and the excessive synthesis of reactive oxygen species. They can be harmful to the pancreatic b-cell, leading to a decrease of insulin secretion and insulin-medicated peripheral glucose uptake^4^. During pregnancy, glucose control is essential to avoid adverse maternal, fetal, or neonatal consequences, such as fetal macrosomia, malformation, and dystocia. Improving our understanding of the metabolic profile and target therapies for its control is a challenge in the pregnant, mainly in those with obesity or diabetes.

There was a significant improvement in subjective sleep quality in the two studies^15,46^ that analyzed it. One performed only ESS^46^, and the other evaluated baseline ESS and PSQI revealing no statistical differences of baseline between the CPAP and control groups^15^. Although subjective sleep quality is an affordable variable to gather, the majority of the selected studies did not issue this data, including all the preeclampsia studies. Possibly, subjective analysis were avoided because pregnancy is a confounding factor for daytime sleepiness and sleep quality^24,25^. They may get worse in a pregnant woman with OSA, possibly due to the growing BMI in the young-adults population^61,62,76^ and their reduction of progesterone levels, the hormone which increases ventilatory drive^25^. Alternatively, sleep quality questionnaires may be validated with a specific normality range for the pregnant population.

Despite the variability in gestational age analysis, all the selected studies^10,12,15,34,35,39,42,46^ have a population with pregnant women under the mean age of 35 years, presenting high BMI or OSA risk factors. Obesity and its consequences are growing concerns worldwide and can lead to a high-risk pregnancy^25^. Three studies were performed by the same group in Australia^35,39,42^, two in the USA, and only two trials were conducted in developing countries^10,12^, so our results may reflect populations of developed countries. Besides, only papers published in English were included; this may cause potential publication bias. Further research may be conducted in developing countries where preeclampsia mortality rates are still high^66^. Hence, according to the current systematic review in pregnant patients with arterial hypertension, diabetes, metabolic syndrome, and dyslipidemia^77,78^, a polysomnography screening test may be considered in prenatal care, especially in women who are symptomatic or have multiple comorbidities^79,80,81,82^. Simplified methods of OSA diagnosis can be more comfortable and accessible, like the controversial types 3 and 4 polysomnography, which do not monitor electroencephalogram derivations. The lack of a standard sleep measurement underestimates arousal index, hypopneas, RERAs, and OSA severity, especially in young women whose respiratory arousal threshold awakes the individual in response to minor changes in respiratory drive without greater hypoxia^83^. These frequent arousals lead to excessive daytime sleepiness, fatigue, insomnia, and other neurocognitive symptoms^84^. The innovative technology needs to be properly investigated. For example, investments in wireless devices for sleep monitoring to provide EEG appear promising. Future research will be essential to establish its diagnostic value and validation to the management of pregnant or non-pregnant populations.

In the last twenty years, few small-sized trials evaluated the CPAP treatment in pregnant women. Hence, to increase our sample and avoid publication bias, RCT, and pre-post studies were included with diverse methods, while other trials without control groups submitted the same group to protocols pre and post CPAP use^34,39,44,46^. Polysomnography was performed before CPAP treatment to diagnose SBD, but none of the studies reported CPAP titration or hours of use per night, which implies long-term outcomes^46^. Mean IAH, RDI, and REI were less than 15 in most studies^10,12,15,34,39,42^, which represents mild OSA severity like expected in young women^85^, but it may decrease CPAP adherence. The CPAP therapy ranged from one or a few days of CPAP treatment^10,15,35,39,42^ to months^12,34,46^, so the knowledge gap could not be fully elucidated, especially fetal and neonatal development. Despite many cardiovascular and BP studies^12,15,34,35,39^, there was a single trial in gestational diabetes women^10^. With regards to the CPAP treatment, given the limitations of published literature focused on therapy for the pregnant women presenting the metabolic syndrome phenotype (hypertension, obesity, gestational diabetes, and OSA), we suggest the following: these women should receive treatment knew to be effective in glucose and BP control, but CPAP may improve BP control, infammatory profile, fetus movement, Apgar score, and reduces preterm birth. Notably, up-to-date studies have failed to show CPAP benefits in birth weight and Apgar score.

The precision medicine concept determines that the success of the therapy is predicted by a phenotype, taking into account its specific physiology and progression of OSA. First of all, each study evaluated the CPAP effects on pregnant population with several comorbidities using different protocols. Moreover, the research outcomes carried out in non-pregnant women may not be applied in preeclampsia, for instance^54,86,87,88^. The AASM classification was applied to the AHI threshold for OSA severity^22^, because it is still undefined in pregnant women. However, further researches should verify if this classification is appropriated and the possible differences according to polysomnography type. Consequently, the implementation of these principles into the management of OSA or snoring during gestation still needs more evidence. Nevertheless, along with the benefits described above, CPAP is a safe and promising treatment for SBD during pregnancy. Future research on pro-infammatory markers that may help to distinguish this entity from others is needed. These goals can only be achieved by cross-institutional collaboration, deep clinical phenotyping from prospectively collected data through collaborative registries, and well-conducted investigations of underlying infammatory mechanisms in a large cohort.

## CONCLUSION

In conclusion, pregnancy is associated with SBD due to physiologic changes, especially during the third trimester, leading to poor sleep quality and several cardiovascular and metabolic complications. Despite the OSA association with pregnancy, in the last 20 years, only eight trials have verified the effects of CPAP therapy on the mother-baby binomial. Maternal comorbidities associated with OSA, such as preeclampsia and gestational diabetes, can be extremely harmful to the woman and developing fetus. Thereby, screening with polysomnography may be considered in pregnant women with high BMI, hypertension, preeclampsia, gestational diabetes, and snoring because OSA still is underdiagnosed. Thus, those diagnosed with OSA or snoring can benefit from CPAP use, especially in BP control during preeclampsia status and in the pancreatic function of women with gestational diabetes. However, the effect of CPAP on fetal and neonatal outcomes still is unclear. CPAP is a safe therapy considered as the standard treatment for SDB during pregnancy, but multicentre randomized trials in a large-sized population are necessary to define high evidence recommendations for clinical practice.

## Figures and Tables

**Table 4.** NIH points for each pre-post study.

NIH GUIDANCE	1	2	3	4	5	6	7	8	9	10	11	12
Edward, 2000					NR							
Guilleminault, 2004					NR							
Guilleminault, 2007					NR							
Blyton, 2013					NR							

Notes: Blue box: YES (low risk); White box: NO (high risk); Gray Box: (unknown risk); CD: Can not determine; NA: Not applicable; NR: Not reported.

1. Was the study question or objective clearly stated? 2. Were eligibility/selection criteria for the study population prespecified and clearly described? 3. Were the participants in the study representative of those who would be eligible for test/service/intervention in the general or clinical population of interest? 4. Were all eligible participants that met the prespecified entry criteria enrolled? 5. Was the sample size sufficiently large to provide confdence in the findings? 6. Was the test/service/intervention clearly described and delivered consistently across the study population? 7. Were the outcome measures prespecified, clearly defined, valid, reliable, and assessed consistently across all study participants? 8. Were the people assessing the outcomes blinded to the participants' exposures/interventions? 9. Was the loss to follow-up after baseline 20% or less? Were those lost to follow-up accounted for in the analysis? 10. Did the statistical methods examine changes in outcome measures from before to after the intervention? Were statistical tests done that provided p-values for the pre-to-post changes? 11. Were outcome measures of interest taken multiple times before the intervention and multiple time after the intervention (i.e., did they use an interrupted time series design)? 12. If the intervention was conducted at a group level (i.g., a hole hospital, a community, etc.) did the statistical analysis take into account the use of individual level data to determine effects at the group level?

**Table 5.** NIH points for each pre-post study.

NIH GUIDANCE	1	2	3	4	5	6	7	8	9	10	11	12	13	14
Blyton, 2004			CD									NR		
Poyares, 2007														
Reid, 2013		NR	NR											
Chirakalwasan, 2018				NR										

Notes: Blue box: YES (low risk); White box: NO (high risk); Gray Box: (unknown risk); CD: Can not determine; NA: Not applicable; NR: Not reported.

1. Was the study described as randomized, a randomized trial, a randomized clinical trial, or an RCT? 2. Was the method of randomization adequate (i.e., use of randomly generated assignment)? 3. Was the treatment allocation concealed (so that assignments could not be predicted)? 4. Were study participants and providers blinded to treatment group assignments? 5. Were the people assessing the outcomes blinded to the participants' group assignments? 6. Were the groups similar at baseline on important characteristics that could affect outcomes (e.g., demographics, risk facts, comorbid conditions)? 7. Was the overall dropout rate from the study at endpoint 20% or lower of the number allocated to treatment? 8. Was the differential dropout rate (between treatment groups) at endpoint 15 percentage points or lower? 9. Was there high adherence to the intervention protocols for each treatment group? 10. Were other interventions avoided or similar in the groups (e.g., similar background treatments)? 11. Were outcomes assessed using valid and reliable measures, implemented consistently across all study participants? 12. Did the authors reported that the sample size was sufficiently large to be able to detect a difference in the main outcome between groups with at least 80% power? 13. Were outcomes reported or subgroups analyzed prespecified (i.e., identified before analyses were conducted)? 14. Were outcomes reported or subgroups analyzed in the group to which they were originally assigned, i.e., did they use an intention-to-treat analysis?
